# Identification of a tertiary lymphoid structure (TLS)-related signature for ovarian cancer prognosis suggests a potential role of STAT5A in TLS maturation

**DOI:** 10.1016/j.gendis.2025.101514

**Published:** 2025-01-04

**Authors:** Jiani Yang, Yue Zhang, Shanshan Cheng, Yanna Xu, Meixuan Wu, Sijia Gu, Mingjun Ma, Yaqian Zhao, Chao Wang, Yu Wang

**Affiliations:** a Department of Gynecology, Shanghai First Maternity and Infant Hospital, School of Medicine, Tongji University, Shanghai 201204, China; b Shanghai Key Laboratory of Maternal Fetal Medicine, Shanghai Institute of Maternal-Fetal Medicine and Gynecologic Oncology, Shanghai First Maternity and Infant Hospital, School of Medicine, Tongji University, Shanghai 200092, China; c Department of Obstetrics and Gynecology, Renji Hospital, School of Medicine, Shanghai Jiaotong University, Shanghai 200127, China

Globally, ovarian cancer (OvCa) is the deadliest gynecological malignancy, which threatens women's health.^1^ Despite innovations in cancer treatments, nearly 70% of OvCa patients still suffered tumor recurrence and poor survival after standard therapy.^1^ Therefore, further research is urgently needed to identify prognostic biomarkers and explore specific mechanisms for OvCa. Tertiary lymphoid structure (TLS), a newly acknowledged form of ectopic lymphoid tissues, plays a crucial role against cancer, though have not been validated in OvCa yet.^2^ Here, we developed and validated a TLS-related gene (TRG) signature to predict drug sensitivity and prognosis for OvCa patients via bioinformatics analysis. Moreover, through PCR and multiplex immunohistochemical analyses, we validated the importance of TLS and the related signature, particularly STAT5A (signal transducer and activator of transcription 5 A), thus assisting clinical decision-making for OvCa precision treatment.

As shown in Figure 1A, we obtained gene expression and clinical characteristics from The Cancer Genome Atlas Program dataset (TCGA-OvCa, *n* = 376, https://portal.gdc.com) as the training cohort, Genotype-Tissue Expression dataset (GTEx, *n* = 180, https://gtexportal.org) as the controls, and International Cancer Genome Consortium dataset (ICGC-OvCa, *n* = 111, https://dcc.icgc.org) as the validation cohort. By searching the Pubmed website (https://pubmed.ncbi.nlm.nih.gov/), we filtered 39 TRGs validated by Fridman and colleagues^3^ and then defined two remarkably different TLS-related patterns, including *Cluster 1* (*n* = 283) and *Cluster 2* (*n* = 91) using the unsupervised clustering method. We proved the great survival advantage of *Cluster 2* over *Cluster 1* (*P* = 0.0451) with Kaplan–Meier curves (Fig. S1). Through the LASSO-COX regression method, we identified an 8-gene signature: risk score = (0.0797) ∗ CCL5 + (0.0653) ∗ CCL8 + (−0.0135) ∗ CCL18 + (−0.0164) ∗ CCL19 + (−0.0827) ∗ CXCL11 + (−0.067) ∗ CXCL13 + (−0.2192) ∗ CD38 + (0.2311) ∗ STAT5A (Fig. 1B). Expression profiles for 8 optimal prognostic TRGs in tumor tissues from TCGA-OvCa cohort (*n* = 376) and normal controls from GTEx cohort (*n* = 180) are presented in Figure S2, and an overview of the 8 TRGs is listed in Table S1. The Kaplan–Meier curves implied that OvCa patients with high STAT5A expression suffered worse overall survival (*P* = 0.021), while patients with high expression of other TRGs had better overall survival (*P* < 0.05; Fig. S2). According to the above formula, we calculated the risk score for patients in the TCGA-OvCa training and ICGC-OvCa validation cohorts, who were then divided into two subgroups using the median as cut-off (Fig. 1C, D, up). We demonstrated that TLS-related signature was associated with prognosis via the Kaplan–Meier analysis in both sets (*P* < 0.05; Fig. 1C, D, bottom). Based on clinical characteristics and the signature, we developed and validated a nomogram to predict overall survival at 1-year, 3-year, and 5-year intervals, with promising optimum performance (*P* < 0.001; Fig. S3).

**Figure 1:**
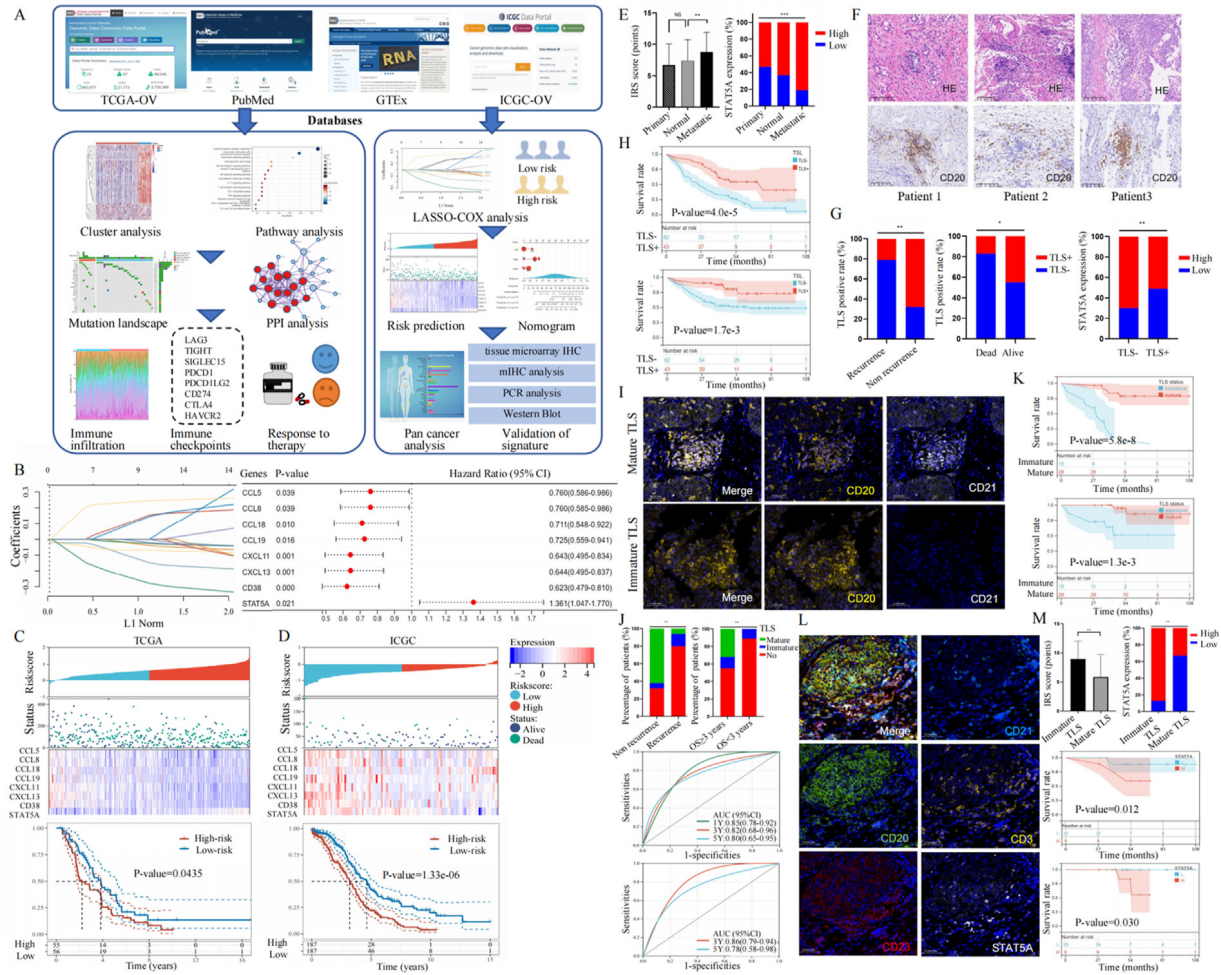
Identification of a TLS-related signature for OvCa prognosis suggests a potential role of STAT5A in TLS maturation. **(A)** The flowchart of study. **(B)** The λ selection (left) and forest diagram (right) for parameter selection through the LASSO-COX algorithm. **(C, D)** The distribution plots (up) and Kaplan–Meier curves (bottom) for TLS-related scores of patients in the TCGA-OvCa training (left) and ICGC-OvCa validation cohorts (right), based on survival status and time. **(E)** Compared with normal controls and primary OvCa lesions, metastatic lesions had up-regulated STAT5A measured by immunohistochemistry staining analysis of tissue microarrays (*n* = 125). **(F)** The representative hematoxylin-eosin and immunohistochemistry staining images of TLSs from three OvCa patients. **(G)** Of the 125 OvCa patients, the TLSs were presented among those with better prognostic outcomes (left). The relationship between TLSs and STAT5A expression (right). **(H)** The Kaplan–Meier curves among 125 OvCa patients classified by TLSs. **(I)** Representative multiplex immunohistochemical images for mature TLS (top) and immature TLS (bottom), among which CD20 (yellow) and CD21 (white) were stained. Nuclei were stained with DAPI (blue). **(J)** Of the 43 TLS-positive OvCa patients, those with mature TLSs were more likely to have a better prognosis, compared with those with immature TLSs (up). The receiver operator characteristic curve analysis (bottom) in 43 TLS-positive OvCa patients stratified by TLS maturity. **(K)** The Kaplan–Meier curves of 43 TLS-positive OvCa patients stratified by TLS maturity. **(L)** Representative multiplex immunohistochemical images of mature TLS, among which CD20 (green), CD23 (red), CD21 (light blue), CD3 (yellow), and STAT5A (white) were stained. Nuclei were stained with DAPI (blue). **(M)** The relationship (up) between TLS maturity and STAT5A among 43 TLS-positive OvCa patients. The Kaplan–Meier curves (bottom) among OvCa patients with TLSs were further stratified by STAT5A expression. TLS, tertiary lymphoid structure; OvCa, ovarian cancer; STAT5A, signal transducer and activator of transcription 5 A.

Through the CIBERSORT algorithm, we evaluated the tumor immune landscape, which indicated up-regulation of T cells, B cells, and macrophages in the low-risk group (*P* < 0.001; Fig. S4A, B). We then applied correlation analysis, demonstrating a negative relationship between M2 macrophages and T follicular helper cells and a positive relationship between CD8^+^ T cells and M1 macrophages (Fig. S4C). Stepwise, we evaluated therapy responses via the tumor immune dysfunction and exclusion (TIDE) algorithm based on the Genomics of Drug Sensitivity in Cancer (GDSC) database, which proved that OvCa individuals with higher TLS scores exhibited less sensitivity toward immune checkpoint blockade therapy (Fig. S4D, E) and more sensitivity toward sorafenib (Fig. S4F). To verify the clinical application of the TLS pattern in various cancers, we applied pan-cancer analysis in TCGA datasets, which proved optimum prognostic performance (Fig. S5).

Especially, we conducted immunohistochemistry staining analysis of the tissue microarrays (OvCa cases = 125), which indicated that metastatic tissues had higher STAT5A expression (Fig. 1E). Table S2 showed that STAT5A expression was not significantly associated with clinical features (*P* > 0.05), while the Kaplan–Meier curve analysis demonstrated that OvCa patients with up-regulated STAT5A suffered worse prognosis (Fig. S6). In Figure 1F, we listed hematoxylin-eosin and immunohistochemistry staining images of TLSs from three representative OvCa patients. We found that OvCa patients without TLS were more likely to suffer recurrence or death (*P* < 0.05; Fig. 1G, left). In Table S3, we estimated the association between TLSs and other features, among which only STAT5A was significantly associated with TLSs (*P* = 0.001). In Table S4, we carried out COX regression analysis to define prognostic indicators, including FIGO stage (hazard ratio = 4.988; 95% confidence interval = 1.992–12.491; *P* = 0.001), STAT5A (hazard ratio = 1.571; 95% confidence interval = 1.131–2.242; *P* = 0.025), and TLS (hazard ratio = 0.110; 95% confidence interval = 0.026–0.471; *P* = 0.003). The results indicated the negative correlation between TLSs and STAT5A expression in OvCa (*P* < 0.05; Fig. 1G, right), and patients without TLS suffered a worse prognosis (*P* < 0.01; Fig. 1H).

Stepwise, we evaluated association between STAT5A and TLS maturity through multiplex immunohistochemical analysis on 43 TLS-positive OvCa patients (Fig. 1I). We found that compared with those with immature TLSs or without TLS, OvCa patients with mature TLSs were more likely to have better prognosis (*P* < 0.01; Fig. 1J, up), while the receiver operator characteristic curve analysis also proved prognostic value of TLS maturity (area under the curve > 0.85; Fig. 1J, bottom). The Kaplan–Meier curves suggested that OvCa individuals with mature TLSs had better prognosis (*P* < 0.001; Fig. 1K). The representative multiplex immunohistochemical images of mature TLSs were stained with CD20, CD23, CD21, CD3, and STAT5A (Fig. 1L). Figure 1M implied that STAT5A was up-regulated among OvCa patients with immature TLSs, and TLS-positive patients with higher STAT5A suffered a worse prognosis (*P* < 0.05).

In brief, we comprehensively analyzed the vital role of TLS pattern in OvCa and identified TRGs to construct an 8-gene prognostic signature via bioinformatics algorithms. We also validated the prognostic signature as a promising tool to predict immune landscape and therapy responses, thus assisting clinical decision-making for OvCa precision medicine. Especially, we explored the importance of STAT5A in OvCa metastasis by influencing TLS maturity, which could provide hints for potential therapeutic targets towards OvCa, though the underlying mechanism still needs further investigation.

## Ethics declaration

The research was approved by the Ethics Committee of Renji Hospital, Shanghai, China (approval number: LY2023-181-B). All patients provided informed consent for the usage of information for research purposes. All authors approved the final version of the manuscript and the submission to *Genes & Diseases*.

## Funding

This study was supported by the 10.13039/501100003399Science and Technology Commission of Shanghai Municipality, China (No. 23YF1433600) and the 10.13039/501100001809National Natural Science Foundation of China (No. 82303652).

## CRediT authorship contribution statement

**Jiani Yang:** Conceptualization, Data curation, Formal analysis, Writing – original draft. **Yue Zhang:** Investigation, Methodology. **Shanshan Cheng:** Methodology, Project administration. **Yanna Xu:** Project administration, Resources. **Meixuan Wu:** Methodology, Project administration. **Sijia Gu:** Resources, Software. **Mingjun Ma:** Supervision, Validation. **Yaqian Zhao:** Validation, Visualization. **Chao Wang:** Validation, Visualization, Writing – review & editing. **Yu Wang:** Funding acquisition, Validation, Visualization, Writing – review & editing.

## Data availability

The data that support the findings of this research are available from the corresponding author upon reasonable request.

## Conflict of interests

The authors declared that the study was conducted in the absence of any financial relationships that might be construed as a potential conflict of interest.
