# Supplementary material for: Identification of a tertiary lymphoid structure (TLS)-related signature for ovarian cancer prognosis suggests a potential role of STAT5A in TLS maturation
Source: Genes Dis. 2025 Jan 4;12(5):101514. doi: 10.1016/j.gendis.2025.101514 (PMC12142517; doi:10.1016/j.gendis.2025.101514)
Supplement: Multimedia component 1 [file mmc1.docx]

**Methods**

**Public databases acquisition and somatic gene mutation analysis**

From the TCGA-OvCa cohort (n=376, https://portal.gdc.com), we obtained RNA-sequencing (RNA-seq) transcriptome data as well as clinical features, up to July 2023. As normal controls, we obtained the RNA-seq data from the GTEx normal cohort (n=180, https://gtexportal.org). As validation, we downloaded the transcriptome data and clinical features from the ICGC-OvCa cohort (n=111, https://dcc.icgc.org). Next, we normalized the RNA-seq transcriptome profiles through the “limma” package. To identify potential TLS-related genes (TRGs), we searched the Pubmed database (https://pubmed.ncbi.nlm.nih.gov) with keywords “tertiary lymphoid structures” and “TLS”. In Figure 1, we graphed the flowchart of the literature.

**Construction and validation of the** **TLS-related signature**

Firstly, to filter potential prognostic TRGs, we applied the Least Absolute Shrinkage and Selection Operator analysis (LASSO, 10-fold cross-validation). We applied the Cox-Regression analysis to enhance model explicability of TLS-related signature. Next, the TLS score of each OvCa individuals were calculated refer to the defined formula of TLS-related signature, using the "glmnet" R package. We then divided OvCa patients into two risk groups by the median TLS score as cut-off. According to the TLS-related score and clinical characteristics, including age, clinical FIGO stage, and pathological grade, we applied both univariable and multivariable Cox-Regression approaches to filter prognostic indicators for OvCa, using the "forestplot" R package. According to the integration of independent prognostic features, we developed a nomogram model to predict overall survival rates at 1-year, 3-year, and 5-year intervals, using the "rms" R package.

**Evolution of tumor immune infiltration and drug sensitivity**

To provide an insight into immune infiltration landscape, we calculated percentage of 22 typical immune cells in tumor microenvironment, according to the CIBERSORT (https://cibersortx.stanford.edu/) algorithm^1^. In order to predict immunotherapy sensitivity, we estimated 8 immune checkpoints expression, including CD274, CTLA4, LAG3, SIGLEC15, HAVCR2, PDCD1LG2, TIGIT, and PDCD1, between 2 TLS-associated risk groups classified by the TLS score-associated signature. Next, we predicted sensitivity towards immune checkpoint blockade (ICB) treatment, through the Tumor Immune Dysfunction and Exclusion (TIDE) algorithm (http://tide.dfci.harvard.edu). We then calculated the half maximal inhibitory concentration values (IC50) for Cisplatin, Bleomycin, Docetaxel, Gemcitabine, Paclitaxel, Veliparib, Sorafenib, and Vinblastine, according to the Genomics of Drug Sensitivity in Cancer (GDSC) dataset (https://www.cancerrxgene.org), using the Ridge Regression method and "pRRophetic" R package.

**Immunohistochemistry and multiplex immunohistochemical evaluation**

After the hydration and wash procedure, we de-waxed samples fixed in formalin solution. In order to block the endogenous peroxidase activity of tissues, we treated the sample sections with the 3% H^2^O^2^ solution, after the microwave antigen retrieval process. Next, we incubated the slides overnight into the Anti-STAT5A Rabbit antibody (Sango Biotech, D220085, 1:100) and the Mouse Anti-Rabbit IgG (Sango Biotech, D110065, 1:100) subsequently. Two experienced pathologists were graded the staining signal intensity of labeled cells via a 4-tier scale (strong 3 points; moderate 2 points; weak 1 point; and absent 0 point) and the signal proportion via a 4-tier scale (>75% 4 points; 51%-75% 3 points; 26%-50% 2 points; 6%-25% 1 point; and 0%-5% 0 point). Refer to the formula: staining signal intensity * signal proportion, we calculated the Immunoreactive score (IRS) of each section^2^.

For multiplex immunohistochemical (mIF) staining, only one antigen was detected in each round, which includes primary antibody incubation, secondary antibody incubation, tyramine signal amplification (TSA) visualization, followed by labeling the next antibody after epitope retrieval and protein blocking as before3. For tumor samples, CD20 (Abclonal, A1793), CD23 (Abclonal, A11436), CD21 (Abclonal, A8407), CD3 (Abclonal, A24060), and STAT5A (Sango Biotech, D220085) were sequentially examined through immunofluorescence (IF) staining. Nuclei were stained with DAPI (blue). The TSA visualization was performed with the Opal 6-Color multiplex IHC kit (Absin, abs50030) following the manufacturer instructions.

**Polymerase Chain Reaction (PCR) analysis**

Referring to manufacturer instructions, total RNA of tumor samples was extracted through the Trizol Reagent (Sango Biotech, B511311). We then reversely transcribed the extracted RNA into corresponding cDNA using the First Strand cDNA Synthesis Kit (Sango Biotech, B300537). Next, we applied PCR analysis using the SYBR Green PCR Mix (Biotech, B110031). The primer sequences for CD38, STAT5A, and GAPDH were as following: Forward: 5′- CAACTCTGTCTTGGCGTCAGT -3′ and Reverse: 5′- CCCATACACTTTGGCAGTCTACA -3′; Forward: 5′- CAGTGGTTTGACGGGGTGAT -3′ and Reverse: 5′- GTCGTGGGCCTGTTGCTTAT -3′; Forward: 5′- CTGGGCTACACTGAGCACC -3′ and Reverse: 5′- AAGTGGTCGTTGAGGGCAATG -3′, respectively. The 2-ΔΔCt method was used to calculate comparative gene expression, setting GAPDH as internal control primer.

**Statistics analysis**

To define prognostic indicators, we applied univariate and multivariate Cox-Regression approaches. We further evaluated prognostic value of TLS score through the Kaplan–Meier (K-M) survival curves, which were analyzed via the Log-rank test. Then, we visualized the Receiver operating characteristics curves (ROC), which were compared via the area under the curve (AUC) value. Categorical and continuous variables were evaluated using the Chi-square test and the T-test, respectively. In our study, we defined log FC = 0.5 and p-value = 0.05 as statistically significant, while all statistical analyses were conducted via the R software (Version 4.0.3).

**Reference**

1. Newman AM, Liu CL, Green MR, et al. Robust enumeration of cell subsets from tissue expression profiles. *Nat Methods.* 2015;12(5):453-457.

2. Specht E, Kaemmerer D, Sanger J, Wirtz RM, Schulz S, Lupp A. Comparison of immunoreactive score, HER2/neu score and H score for the immunohistochemical evaluation of somatostatin receptors in bronchopulmonary neuroendocrine neoplasms. *Histopathology.* 2015;67(3):368-377.

3. He M, He Q, Cai X, et al. Intratumoral tertiary lymphoid structure (TLS) maturation is influenced by draining lymph nodes of lung cancer. *Journal For Immunotherapy of Cancer.* 2023;11(4).
