# Supplementary material for: Identification of a tertiary lymphoid structure (TLS)-related signature for ovarian cancer prognosis suggests a potential role of STAT5A in TLS maturation
Source: Genes Dis. 2025 Jan 4;12(5):101514. doi: 10.1016/j.gendis.2025.101514 (PMC12142517; doi:10.1016/j.gendis.2025.101514)

**Figure S1. Identification of TLS-related pattern and somatic alteration landscape.** (A) The consensus matrices heatmap of TCGA-OvCa patients refer to the TLS-related genes through the unsupervised clustering method. (B) The Consensus clustering cumulative distribution function plot (CDF, left) and related change in area under CDF curve (CDF Delta area, right) of TLS-related pattern. (C) The Principal Component Analysis (PCA) analysis of TLS-related subgroups among TCGA-OvCa individuals. (D) We evaluated prognostic value of the TLS-related pattern among TCGA-OvCa cohort through the Kaplan–Meier (K-M) survival curves, which were analyzed via the Log-rank test. (E) The mutation frequency and Somatic cell copy number alternation (SCNA) in the TCGA-OvCa cohort. (F) The protein-protein interaction network (PPI) of 39 TLS-related genes through Metascape website (https://metascape.org). (G) The KEGG and (H) GO pathways enrichment analysis according to defined TLS-related genes.


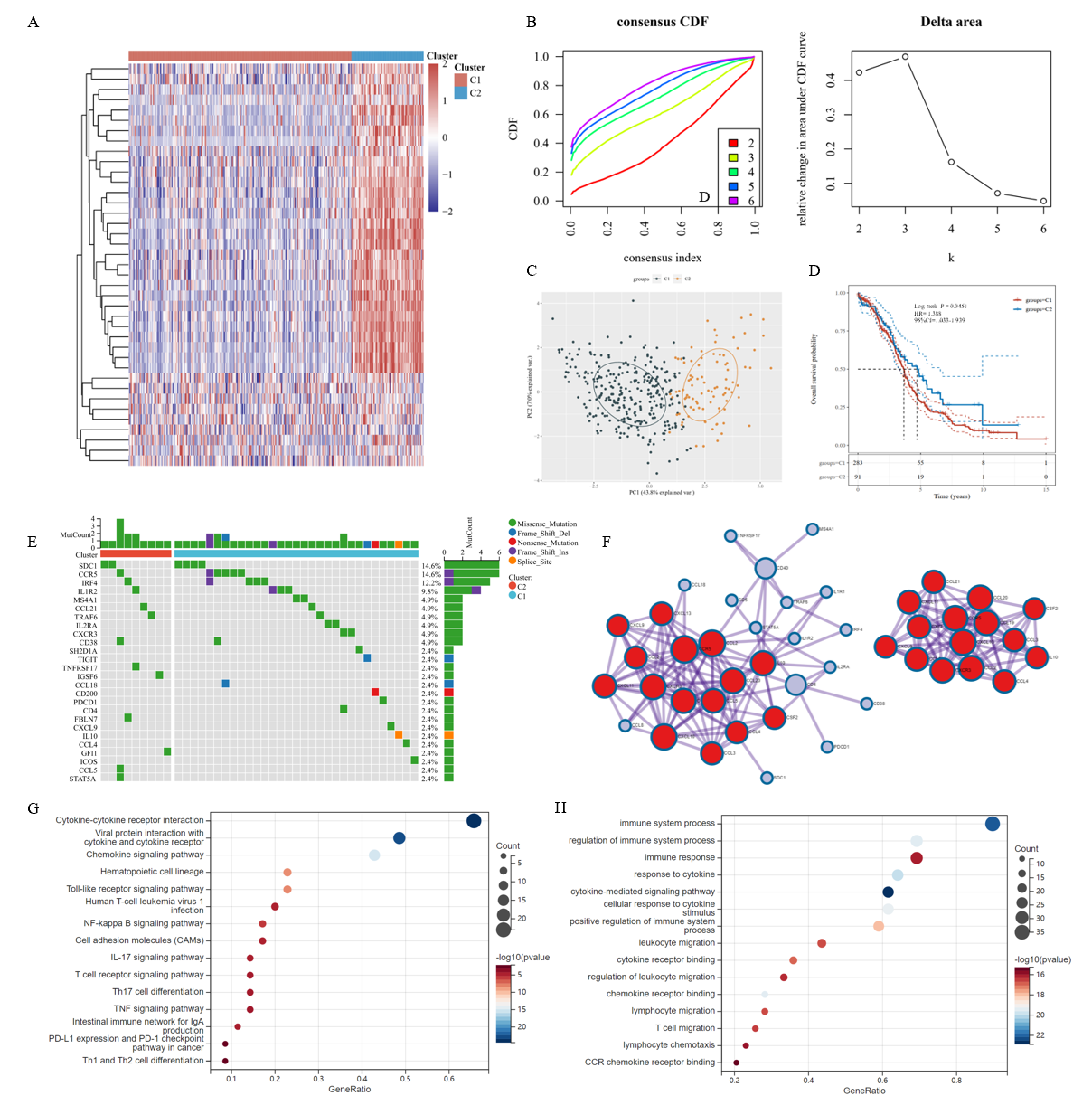

Supplement: Multimedia component 2 [file mmc2.docx]
