# Supplementary material for: Identification of a tertiary lymphoid structure (TLS)-related signature for ovarian cancer prognosis suggests a potential role of STAT5A in TLS maturation
Source: Genes Dis. 2025 Jan 4;12(5):101514. doi: 10.1016/j.gendis.2025.101514 (PMC12142517; doi:10.1016/j.gendis.2025.101514)

**Figure S2. Establishment of the Tertiary Lymphoid Structure (TLS)-related signature in ovarian cancer (OvCa).** (A) Expression profile for 8 optimal prognostic TLS-related genes (TRGs), including CCL5, CCL8, CCL18, CCL19, CXCL11, CXCL13, CD38, and STAT5A, in tumor tissues from the TCGA-OvCa cohort (n=376, https://portal.gdc.com) and normal controls from the GTEx cohort (n=180, https://gtexportal.org). (B) Sankey plot graphed the relationship between TLS-related signature and corresponding clinical characteristics, including clinical FIGO stage, age, race, pathological grade, and survival status. (C) Based on the median as cut-off value, we evaluated prognostic value of the filtered 8 TRGs among TCGA-OvCa cohort through the Kaplan–Meier (K-M) survival curves, which were analyzed via the Log-rank test.


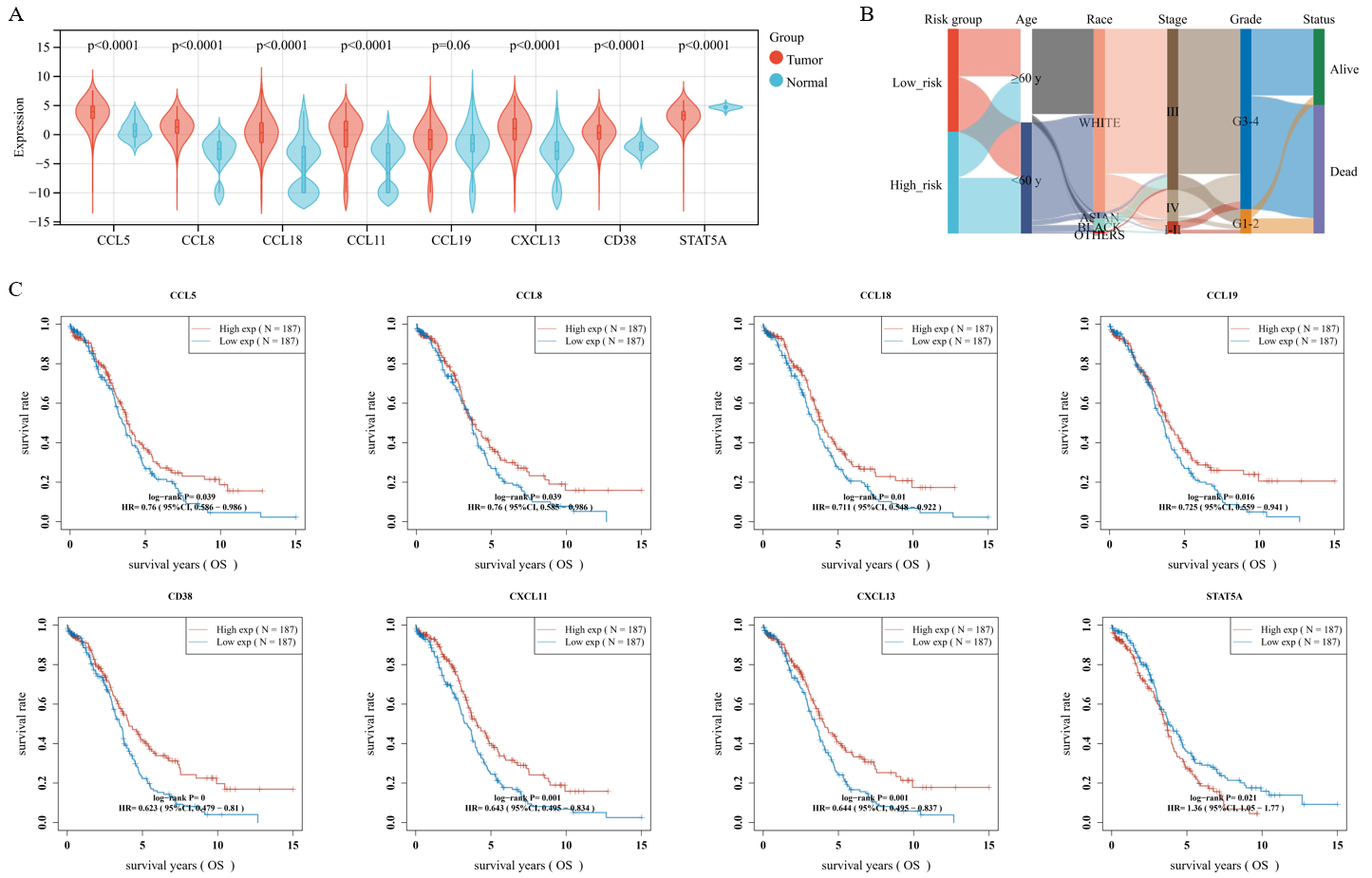

Supplement: Multimedia component 3 [file mmc3.docx]
