# Supplementary material for: Identification of a tertiary lymphoid structure (TLS)-related signature for ovarian cancer prognosis suggests a potential role of STAT5A in TLS maturation
Source: Genes Dis. 2025 Jan 4;12(5):101514. doi: 10.1016/j.gendis.2025.101514 (PMC12142517; doi:10.1016/j.gendis.2025.101514)

**Figure S3. Construction and validation of TLS score-associated nomogram among ovarian cancer (OvCa) patients.** The forest diagrams of (A) univariate and (B) multivariate Cox-Regression approaches for OvCa prognosis, according to the TLS-related score and clinical characteristics, including age, clinical FIGO stage, and pathological grade. (C) The quantitative prognostic nomogram to predict OS at 1-year, 3-year, and 5-year intervals, based on the TLS-related signature and clinical indicators. (D) The calibration plots graphed the consistency between the observed and predicted OS among OvCa patients at 1-year, 3-year, and 5-year intervals (top, middle, and bottom). The K-M curves (left) and time-dependent ROC analysis (right) for individuals stratified by the TLS score-associated nomogram, among (E) TCGA-OvCa training and (F) ICGC-OvCa validation cohorts.


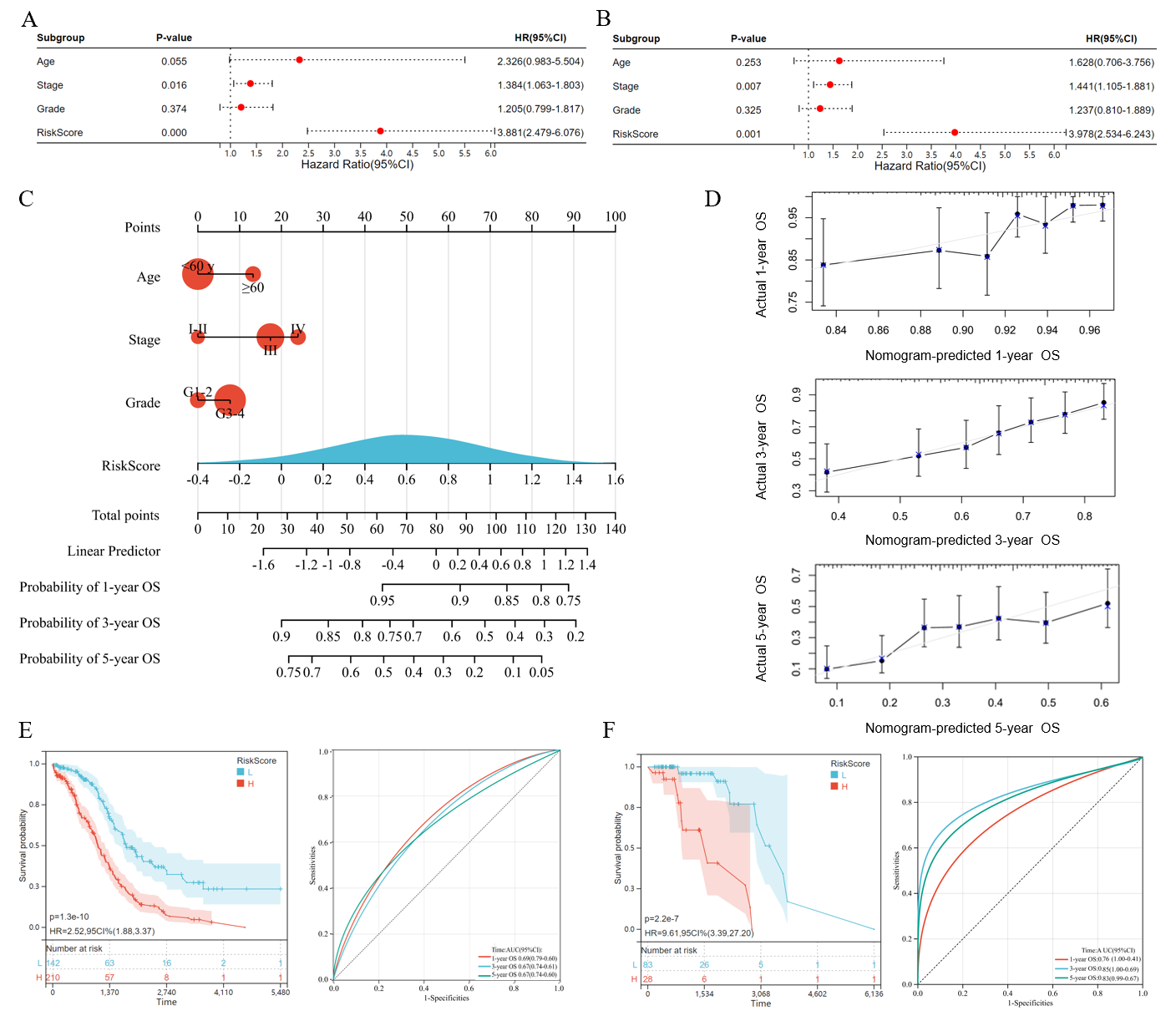

Supplement: Multimedia component 4 [file mmc4.docx]
