# Supplementary material for: Identification of a tertiary lymphoid structure (TLS)-related signature for ovarian cancer prognosis suggests a potential role of STAT5A in TLS maturation
Source: Genes Dis. 2025 Jan 4;12(5):101514. doi: 10.1016/j.gendis.2025.101514 (PMC12142517; doi:10.1016/j.gendis.2025.101514)

**Figure S4. Tumor immune microenvironment landscape and drug sensitivity related to the TLS score-associated signature.** (A) Boxplots showed proportion of 22 typical immune cells infiltrated in OvCa tumor tissues through the CIBERSORT analysis, among patients stratified by the TLS score. (B) Violin diagrams compared distribution of 22 infiltrated immune cells, through the CIBERSORT analysis, among patients stratified by the TLS score. (C) The heat diagram graphed correlation matrix of 22 immune cells infiltrated in ovarian cancer (OvCa) patients. (D) Boxplots of expression profile for eight typical immune checkpoints expression, including CD274, CTLA4, LAG3, SIGLEC15, HAVCR2, PDCD1LG2, TIGIT, and PDCD1, between two TLS-related risk groups. (E) Prediction of sensitivity towards immune checkpoint blockade (ICB) treatments via the Tumor Immune Dysfunction and Exclusion (TIDE) algorithm. (F) Violin diagrams of the estimated IC50 values for Cisplatin, Bleomycin, Docetaxel, Gemcitabine, Paclitaxel, Veliparib, Sorafenib, and Vinblastine, according to the Genomics of Drug Sensitivity in Cancer (GDSC) dataset.

*p-value < 0.05; **p-value < 0.01; ****p-value < 0.0001


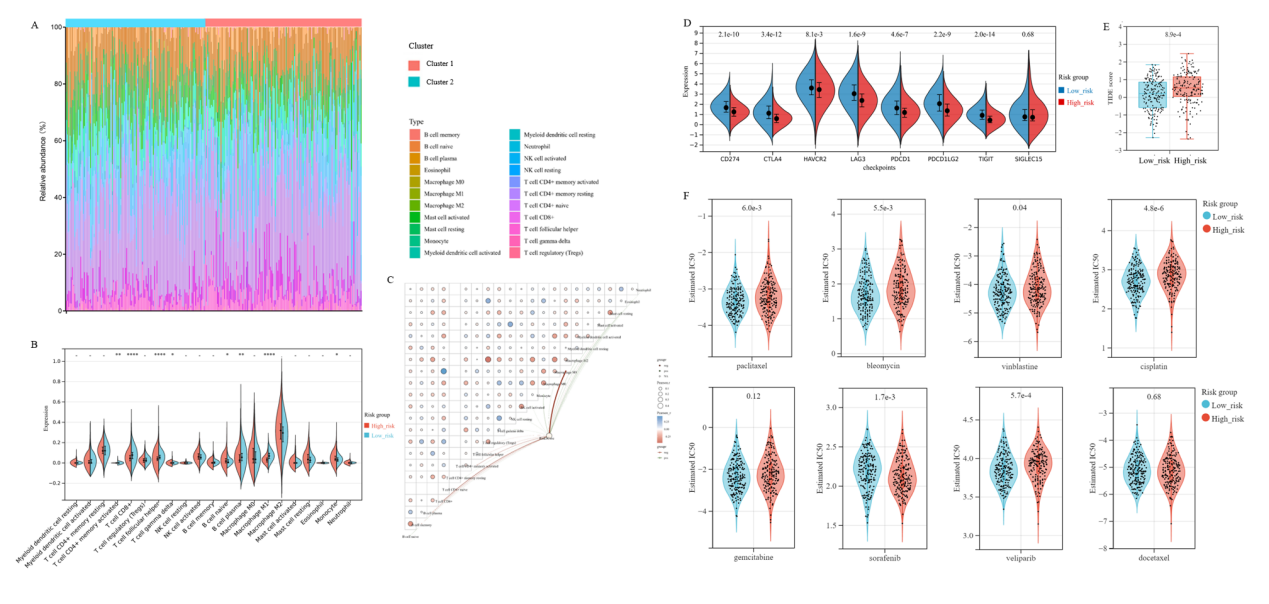

Supplement: Multimedia component 5 [file mmc5.docx]
