# Supplementary material for: Identification of a tertiary lymphoid structure (TLS)-related signature for ovarian cancer prognosis suggests a potential role of STAT5A in TLS maturation
Source: Genes Dis. 2025 Jan 4;12(5):101514. doi: 10.1016/j.gendis.2025.101514 (PMC12142517; doi:10.1016/j.gendis.2025.101514)

**Figure S5. Pan-cancer analysis of TLS-associated signature.** Radar plots showed gene expression of (A) STAT5A and (B) CD38, compared among the TCGA pan-cancer cohorts. (C) Violin plots graphed TLS score distribution of tumor tissues and normal controls in pan-cancer. (D) Forest charts distinguished prognostic value of TLS-associated signature via Cox-Regression algorithm in pan-cancer. (E) The K-M survival curves for Ovarian serous cystadenocarcinoma (OV), Glioma (GBMLGG), Low-grade glioma (LGG), Skin cutaneous melanoma (SKCM), Cervical squamous cell carcinoma (CESC), Breast invasive carcinoma (BRCA), Pan-kidney (KIPAN), and Kidney renal papillary cell carcinoma (KIRP) in the TCGA cohorts, which were classified by the TLS score and analyzed via the Log-rank test. (F) Association analysis between TLS score and tumor immune infiltration in pan-cancer, estimated through CIBERSORT algorithm.


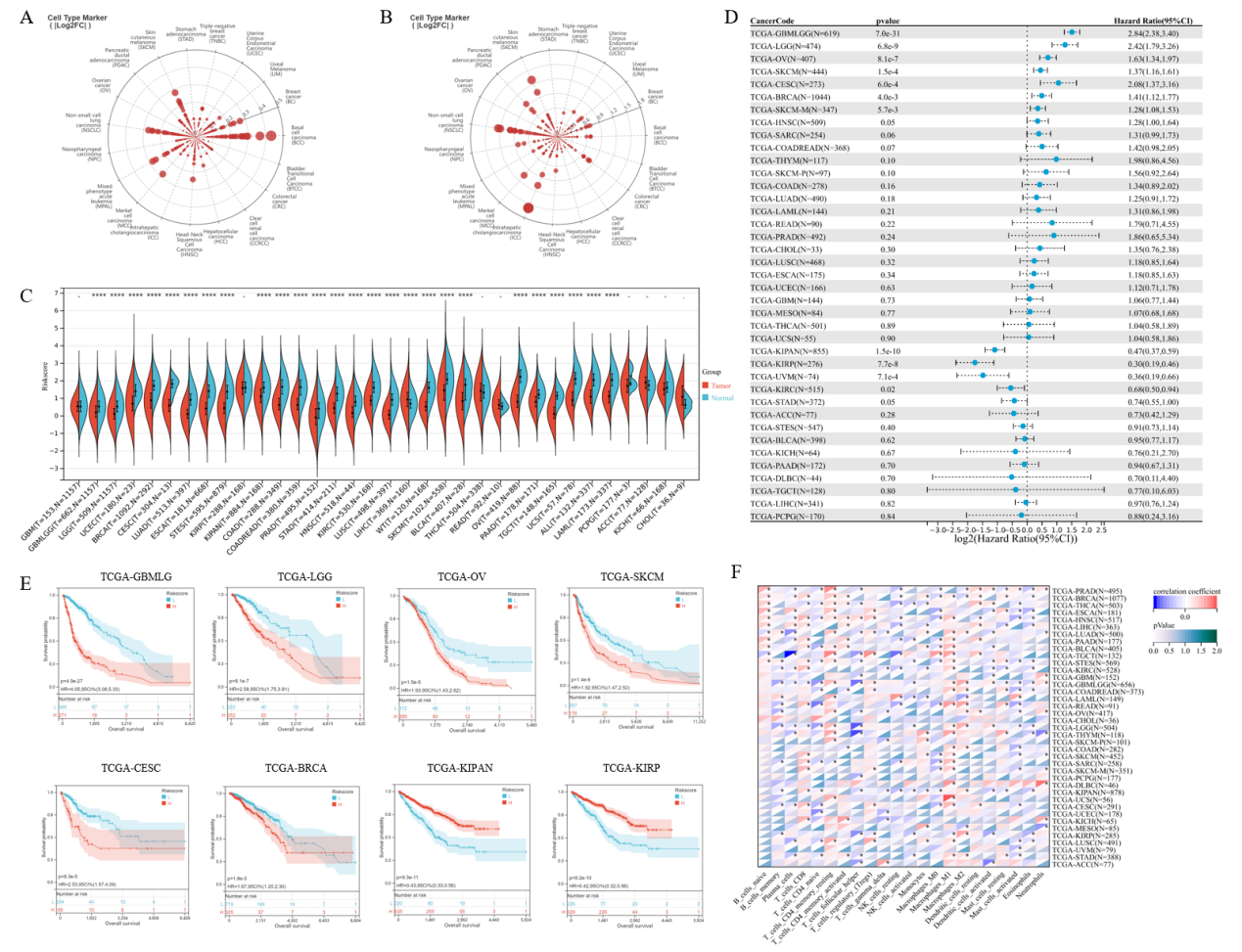

Supplement: Multimedia component 6 [file mmc6.docx]
