# Supplementary material for: Identification of a tertiary lymphoid structure (TLS)-related signature for ovarian cancer prognosis suggests a potential role of STAT5A in TLS maturation
Source: Genes Dis. 2025 Jan 4;12(5):101514. doi: 10.1016/j.gendis.2025.101514 (PMC12142517; doi:10.1016/j.gendis.2025.101514)

**Figure S6. Aberrant up-regulation of** **STAT5A related to metastasis and Tertiary Lymphatic Structure (TLS) in Ovarian Cancer (OvCa).** (A) Representative IHC staining images of STAT5A in primary OvCa lesions, metastatic OvCa lesions, and normal controls. (B) 5 pairs of representative IHC staining images for primary and metastatic OvCa lesions. (C) The K-M survival analysis among OvCa individuals for overall survival (up) and recurrence-free survival (bottom), who were classified by STAT5A expression and analyzed via the Log-rank test. (D) Representative mIHC images of immature TLS, among which CD20 (green), CD23 (red), CD21 (light blue), CD3 (yellow), and STAT5A (white) were stained. Nuclei were stained with DAPI(blue).


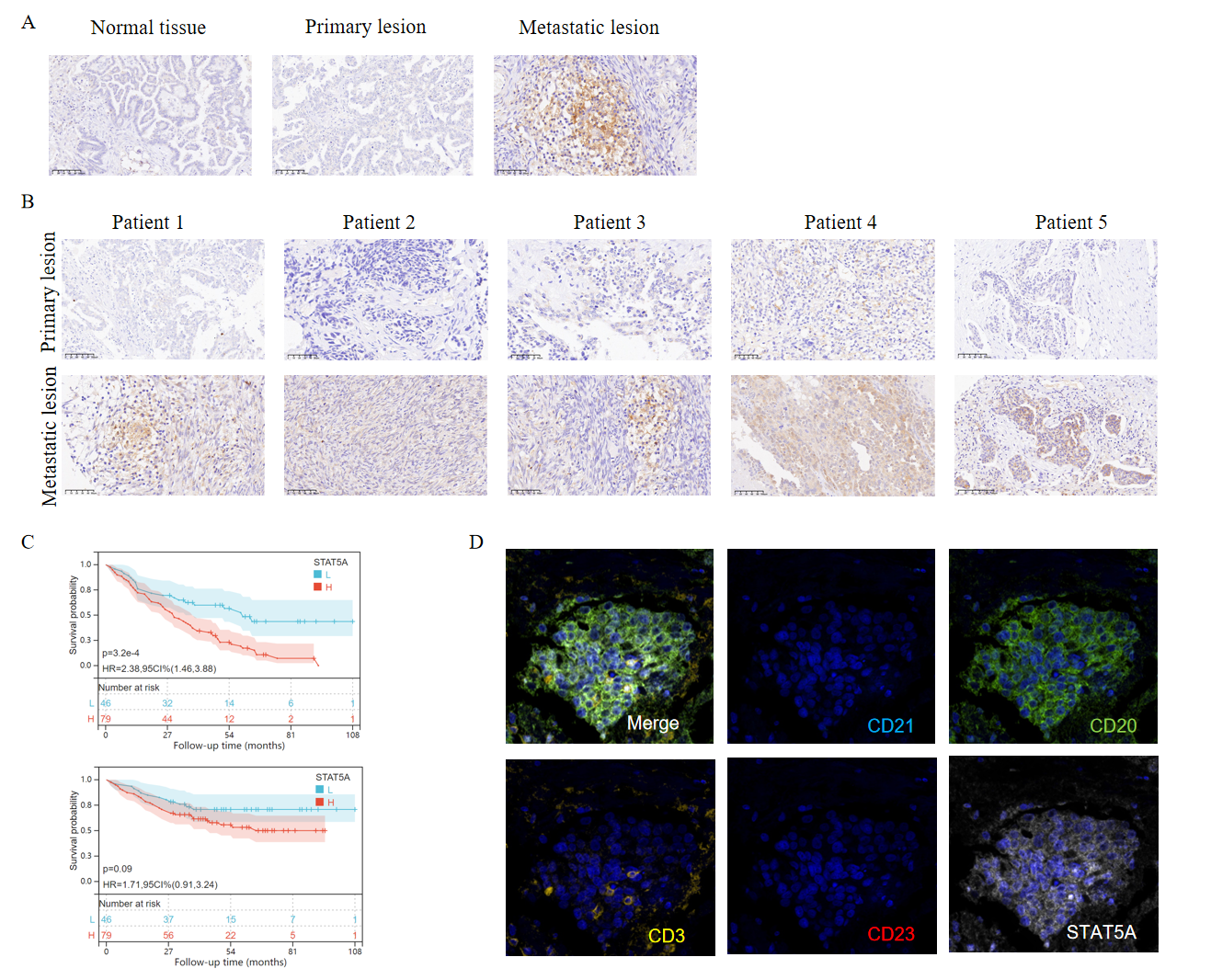

Supplement: Multimedia component 7 [file mmc7.docx]
