# Supplementary material for: Identification of a tertiary lymphoid structure (TLS)-related signature for ovarian cancer prognosis suggests a potential role of STAT5A in TLS maturation
Source: Genes Dis. 2025 Jan 4;12(5):101514. doi: 10.1016/j.gendis.2025.101514 (PMC12142517; doi:10.1016/j.gendis.2025.101514)
Supplement: Multimedia component 8 [file mmc8.docx]

**Table S1:** **The overview of 8 Tertiary Lymphoid Structure (TLS)-related signature genes**^1-11^**.**

| **Symbol** | **Gene Name** | **Summary of the function in ovarian cancer (OvCa)** | **Refer** |
| --- | --- | --- | --- |
| CCL5 | C-C Motif Chemokine Ligand 5 | CCL5, a chemotactic ligand enriched in NK cells and T cells, could mediate OvCa cell survival through the interaction with SDC4. Tumor stem cells enhance tumor progression and promote immune privilege via CCL5 in OvCa. | ^1;2^ |
| CCL8 | C-C Motif Chemokine Ligand 8 | CCL8, a member of a conserved chemokine cluster known as CC cluster, has tumor-promoting activity in breast cancer, by recruiting M2 Macrophages. Unknown in OvCa. | ^3^ |
| CCL18 | C-C Motif Chemokine Ligand 18 | CCL18, a chemokine with an important role in chemokine-mediated cancer metastasis, could promote epithelial ovarian cancer metastasis through the mTORC2 signaling pathway | ^4^ |
| CCL19 | C-C Motif Chemokine Ligand 19 | CCL19, a lymphocyte-migrating chemokine, could suppress metastasis of murine ovarian cancer by activating local immunity through embryonic endothelial progenitor cells. | ^5^ |
| CXCL11 | C-X-C Motif Chemokine Ligand 11 | CXCL11, a fibroblast-secreted chemokine, could promote OvCa proliferation and migration via the interaction with chemokine receptor CXCR3. Moreover, the activation of the CXCR7/CXCL11 axis under ERα control could promote metastatic behavior of OvCa cells by inducing epithelial-mesenchymal transition. | ^6;7^ |
| CXCL13 | C-X-C Motif Chemokine Ligand 13 | In the early phase of tertiary lymphoid structures, CXCL13 could produce CD4+ T cells accumulate in OvCa tissues and facilitate coordinated antitumor response. | ^8^ |
| CD38 | CD38 | CD38, a multifunctional glycoprotein as an immune receptor and ectoenzyme, could predict favorable prognosis and immune cell infiltration, especially the infiltration of activated B cells, CD4+T cells, and CD8+T cells in the OvCa microenvironment. | ^9^ |
| STAT5A | Signal Transducer And Activator Of Transcription 5A | STAT5A, a member of the STAT family, could reprogram fatty acid metabolism and promote tumorigenesis by binding to the promoter of FABP5 in gastric cancer. Moreover, STAT5A activation could interact with the T cell receptor complex and stimulate T cell proliferation, though unknown in OvCa metastasis. | ^10;11^ |

Reference:

1. You Y, Li Y, Li M, et al. Ovarian cancer stem cells promote tumour immune privilege and invasion via CCL5 and regulatory T cells. *Clinical and Experimental Immunology.* 2018;191(1):60-73.

2. Kim S, Han Y, Kim SI, et al. Computational modeling of malignant ascites reveals CCL5-SDC4 interaction in the immune microenvironment of ovarian cancer. *Molecular Carcinogenesis.* 2021;60(5):297-312.

3. Farmaki E, Kaza V, Chatzistamou I, Kiaris H. CCL8 Promotes Postpartum Breast Cancer by Recruiting M2 Macrophages. *IScience.* 2020;23(6):101217.

4. Wang Q, Tang Y, Yu H, et al. CCL18 from tumor-cells promotes epithelial ovarian cancer metastasis via mTOR signaling pathway. *Molecular Carcinogenesis.* 2016;55(11):1688-1699.

5. Hamanishi J, Mandai M, Matsumura N, et al. Activated local immunity by CC chemokine ligand 19-transduced embryonic endothelial progenitor cells suppresses metastasis of murine ovarian cancer. *Stem Cells (Dayton, Ohio).* 2010;28(1):164-173.

6. Benhadjeba S, Edjekouane L, Sauvé K, Carmona E, Tremblay A. Feedback control of the CXCR7/CXCL11 chemokine axis by estrogen receptor α in ovarian cancer. *Molecular Oncology.* 2018;12(10):1689-1705.

7. Lau T-S, Chung TK-H, Cheung T-H, et al. Cancer cell-derived lymphotoxin mediates reciprocal tumour-stromal interactions in human ovarian cancer by inducing CXCL11 in fibroblasts. *The Journal of Pathology.* 2014;232(1):43-56.

8. Ukita M, Hamanishi J, Yoshitomi H, et al. CXCL13-producing CD4+ T cells accumulate in the early phase of tertiary lymphoid structures in ovarian cancer. *JCI Insight.* 2022;7(12).

9. Zhu Y, Zhang Z, Jiang Z, Liu Y, Zhou J. CD38 Predicts Favorable Prognosis by Enhancing Immune Infiltration and Antitumor Immunity in the Epithelial Ovarian Cancer Microenvironment. *Front Genet.* 2020;11:369.

10. Welte T, Leitenberg D, Dittel BN, et al. STAT5 interaction with the T cell receptor complex and stimulation of T cell proliferation. *Science (New York, NY).* 1999;283(5399):222-225.

11. Dong SR, Ju XL, Yang WZ. STAT5A reprograms fatty acid metabolism and promotes tumorigenesis of gastric cancer cells. *European Review For Medical and Pharmacological Sciences.* 2019;23(19):8360-8370.
