# Supplementary material for: Identification of a tertiary lymphoid structure (TLS)-related signature for ovarian cancer prognosis suggests a potential role of STAT5A in TLS maturation
Source: Genes Dis. 2025 Jan 4;12(5):101514. doi: 10.1016/j.gendis.2025.101514 (PMC12142517; doi:10.1016/j.gendis.2025.101514)
Supplement: Multimedia component 9 [file mmc9.docx]

**Table S2.** **The correlation between STAT5A expression and clinicopathological features of 125 OvCa patients.**

| **Characteristic** | **No. of patients** | **STAT5A expression** | | **p-value** |
| --- | --- | --- | --- | --- |
|  |  | **Low**  **(IRS score<8)** | **High**  **(IRS score≥8)** |  |
| **Age (n,%)** |  |  |  | 0.515 |
| **<55 years** | 56(44.8%) | 21(16.8%) | 35(28.0%) | - |
| **≥55 years** | 69(55.2%) | 25(20.0%) | 44(35.2%) | - |
| **FIGO stage (n,%)** |  |  |  | 0.343 |
| **I-II** | 45(36.0%) | 15(12.0%) | 30(24.0%) | - |
| **III-IV** | 80(64.0%） | 31(24.8%) | 49(39.2%) | - |
| **Pathology grade (n,%)** |  |  |  | 0.406 |
| **I-II** | 54(43.2%) | 21(16.8%) | 33(26.4%) | - |
| **III** | 71(56.8%) | 25(20.0%) | 46(36.8%) | - |
| **Histology type (n,%)** |  |  |  | 0.759 |
| **Serous** | 78(62.4%) | 30(24.0%) | 48(38.4%) | - |
| **Mucous** | 11(8.8%) | 4(3.2%) | 7(5.6%) | - |
| **Endometrioid** | 14(11.2%) | 6(4.8%) | 8(6.4%) | - |
| **Other types** | 22(17.6%) | 6(4.8%) | 16(12.8%) | - |
| **Tumor diameter (n,%)** |  |  |  | 0.269 |
| **<10 cm** | 62(49.6%) | 26(20.8%) | 36(28.8%) | - |
| **≥10 cm** | 63(50.4%) | 20(16.0%) | 43(34.4%) | - |
| **Serum CA125 (n, %)** |  |  |  | 0.140 |
| **<35 U/ml** | 20(16.0%) | 10(8.0%) | 10(8.0%) | - |
| **≥35 U/ml** | 105(84.0%) | 36(28.8%) | 69(55.2%) | - |

Abbreviation: FIGO stage, Federation International of Gynecology and Obstetrics stage
