# Supplementary material for: Identification of a tertiary lymphoid structure (TLS)-related signature for ovarian cancer prognosis suggests a potential role of STAT5A in TLS maturation
Source: Genes Dis. 2025 Jan 4;12(5):101514. doi: 10.1016/j.gendis.2025.101514 (PMC12142517; doi:10.1016/j.gendis.2025.101514)
Supplement: Multimedia component 10 [file mmc10.docx]

**Table S3.** **The correlation between Tertiary Lymphatic Structure (TLS) and clinicopathological features of 125 OvCa patients.**

| **Characteristic** | **No. of patients** | **TLS status** | | | **p-value** |
| --- | --- | --- | --- | --- | --- |
|  |  | **No** | **Immature** | **Mature** |  |
| **Age (n,%)** |  |  |  |  | 0.579 |
| **<55 years** | 56(44.8%) | 39(31.2%) | 5(4.0%) | 12(9.6%) | - |
| **≥55 years** | 69(55.2%) | 43(34.4%) | 10(8.0%) | 16(12.8%) | - |
| **FIGO stage (n,%)** |  |  |  |  | 0.084 |
| **I-II** | 45(36.0%) | 25(20.0%) | 9(7.2%) | 11(8.8%) | - |
| **III-IV** | 80(64.0%） | 57(45.6%) | 6(4.8%) | 17(13.6%) | - |
| **Pathology grade (n,%)** |  |  |  |  | 0.393 |
| **I-II** | 54(43.2%) | 39(31.2%) | 5(4.0%) | 10(8.0%) | - |
| **III** | 71(56.8%) | 43(34.4%) | 10(8.0%) | 18(14.4%) | - |
| **Histology type (n,%)** |  |  |  |  | 0.079 |
| **Serous** | 78(62.4%) | 57(45.6%) | 6(4.8%) | 15(12.0%) | - |
| **Mucous** | 11(8.8%) | 5(4.0%) | 4(3.2%) | 2(1.6%) | - |
| **Endometrioid** | 14(11.2%) | 9(7.2%) | 1(0.8%) | 4(3.2%) | - |
| **Other types** | 22(17.6%) | 11(8.8%) | 4(3.2%) | 7(5.6%) | - |
| **Tumor diameter (n,%)** | |  |  |  | 0.084 |
| **<10 cm** | 62(49.6%) | 37(29.6%) | 6(4.8%) | 19(15.2%) | - |
| **≥10 cm** | 63(50.4%) | 45(36.0%) | 9(7.2%) | 9(7.2%) | - |
| **Serum CA125 (n, %)** |  |  |  |  | 0.668 |
| **<35 U/ml** | 20(16.0%) | 12(9.6%) | 2(1.6%) | 6(4.8%) | - |
| **≥35 U/ml** | 105(84.0%) | 70(56.0%) | 13(10.4%) | 22(17.6%) | - |
| **STAT5A expression (n, %)** | |  |  |  | 0.001 |
| **Low (IRS <8)** | 46(36.8%) | 25(20.0%) | 2(1.6%) | 19(15.2%) | - |
| **High (IRS≥8)** | 79(63.2%) | 57(45.6%) | 13(10.4%) | 9(7.2%) | - |

Abbreviation: FIGO stage, Federation International of Gynecology and Obstetrics stage
