# Supplementary material for: Identification of a tertiary lymphoid structure (TLS)-related signature for ovarian cancer prognosis suggests a potential role of STAT5A in TLS maturation
Source: Genes Dis. 2025 Jan 4;12(5):101514. doi: 10.1016/j.gendis.2025.101514 (PMC12142517; doi:10.1016/j.gendis.2025.101514)
Supplement: Multimedia component 11 [file mmc11.docx]

**Table S4. Univariate and multivariate Cox Regression analysis of prognostic factors of the 125 ovarian cancer (OvCa) patients.**

| **Characteristic** | **Univariate Analysis** | | **Multivariate Analysis** | |
| --- | --- | --- | --- | --- |
|  | **HR (****95% CI)** | **P-value** | **HR (95% CI)** | **P-value** |
| **Age** |  |  |  |  |
| **<55 years** | Reference | - | Reference | - |
| **≥55 years** | 1.052(0.595-1.862) | 0.861 | 0.908(0.501-1.648) | 0.752 |
| **FIGO stage** |  |  |  |  |
| **I-II** | Reference | - | Reference | - |
| **III-IV** | 4.238(1.897-9.471) | 0.001 | 4.988(1.992-12.491) | 0.001 |
| **Pathology grade** |  |  |  |  |
| **I-II** | Reference | - | Reference | - |
| **III** | 0.727(0.413-1.282) | 0.271 | 0.787(0.409-1.513) | 0.472 |
| **Histology type** |  | 0.850 |  | 0.563 |
| **Serous** | Reference | - | Reference | - |
| **Mucous** | 0.591(0.181-1.932) | 0.384 | 1.521(0.363-6.374) | 0.566 |
| **Endometrioid** | 0.875(0.341-2.245) | 0.780 | 1.415(0.530-3.779) | 0.489 |
| **Other types** | 0.977(0.450-2.122) | 0.953 | 1.844(0.731-4.650) | 0.195 |
| **Tumor diameter** |  |  |  |  |
| **<10 cm** | Reference | - | Reference | - |
| **≥10 cm** | 1.253(0.710-2.211) | 0.437 | 1.184(0.650-2.154) | 0.581 |
| **Serum CA125** |  |  |  |  |
| **<35 U/ml** | Reference | - | Reference | - |
| **≥35 U/ml** | 2.680(0.960-7.486) | 0.060 | 1.731(0.535-5.600) | 0.360 |
| **TLS expression** |  |  |  |  |
| **Negative** | Reference | - | Reference | - |
| **Positive** | 0.110(0.027-0.457) | 0.002 | 0.110(0.026-0.471) | 0.003 |
| **STAT5A expression** |  |  |  |  |
| **Low (IRS score<8)** | Reference | - | Reference | - |
| **High (IRS score≥8)** | 1.903(1.707-3.227) | 0.010 | 1.571(1.131-2.242) | 0.025 |

Abbreviation: HR, hazard ratio; 95% CI, 95% confidence interval; FIGO stage, Federation of International of Gynecologists and Obstetricians stage; TLS, Tertiary Lymphatic Structure
